# Cytomegalovirus infection: friend or foe in rheumatoid arthritis?

**DOI:** 10.1186/s13075-020-02398-3

**Published:** 2021-01-07

**Authors:** Jean-Luc Davignon, Bernard Combe, Alain Cantagrel

**Affiliations:** 1 Centre de Physiopathologie Toulouse Purpan, U.1043 INSERM, CNRS, CHU Purpan, BP 3028, 31024 Toulouse cedex 3, France; 2 Centre de Rhumatologie, CHU de Toulouse, Toulouse, France; 3 Lapeyronie Hospital, Montpellier I University, UMR, 5535 Montpellier, France; 4 Faculté de Médecine, Université Paul Sabatier Toulouse, Toulouse, France

**Keywords:** Rheumatoid arthritis, Cytomegalovirus, Bone erosion, Inflammation, QKI5, Osteoclast

## Abstract

Human cytomegalovirus (HCMV) is a β-herpesvirus that causes inflammation and remains for life in a latent state in their host. HCMV has been at the center of many hypotheses regarding RA.

We have recently shown that HCMV infection impairs bone erosion through the induction of the mRNA-binding protein QKI5. Latently infected RA patients display a slower progression of bone erosion in patients from a national cohort. Our observations question the possible association between HCMV and the pathophysiology of RA. In this review, we examine the possibility that HCMV may be an aggravating factor of inflammation in RA while protecting from bone erosion. We also assess its relationship with other pathogens such as bacteria causing periodontitis and responsible for ACPA production.

This review thus considers whether HCMV can be regarded as a friend or a foe in the pathogenesis and the course of RA.

## Introduction

Rheumatoid arthritis (RA) is a systemic disease that presents with articular inflammation and eventually deformations and destruction in the absence of treatment [1]. RA is frequent in every part of the world with similar prevalence ranging from 0.5 to 1% [2].

The infectious origin of autoimmune diseases such as RA has been the topic of many studies. Both viral and bacterial origins have been proposed [3].

Epidemiological relationship between bacterial infection of the periodontium such as *Porphyromonas gingivalis* (PG) and the frequency of RA has suggested a pathophysiological link between periodontal disease (PD) and RA, although some studies dispute there could be a link, as reviewed in Potempa et al. [3]. Antibody response to proteins modified by deimination, the so-called anti-citrullinated protein antibodies (ACPA), has been used for years as a tool for diagnosis [4] and now as an etiological mechanism. Thus, the contribution of periodontal bacteria has been proposed among the possible causes of RA.

Since herpesviruses remain for life in a latent state in their host, they have also been at the center of many hypotheses regarding autoimmune diseases but no definitive conclusion has been reached regarding their role in RA.

This review focuses on the possible association between cytomegalovirus, a β-herpesvirus, and the pathophysiology of RA. This is based on our observation that, in the large national “ESPOIR” cohort, patients seropositive for HCMV were protected from bone erosion [5]. We thus present different views on the role of HCMV in RA, its relationship with periodontium bacteria, and its consequence on bone erosion. We thus discuss whether HCMV can be considered as a friend or a foe in the pathogenesis and the course of RA.

## Bacteria in the pathogenicity of RA

The discovery of anti-citrullinated protein antibodies (ACPA) in the serum of RA patients, as reviewed in [4], has revolutionized the diagnosis and subsequently the etiopathogenesis of the disease. The presence of ACPA is very specific of RA and is part of the elements of the diagnosis [6]. They define a population of RA patients with higher severity and stronger bone erosion. Citrullination is due to the modification of Arginin residues into Citrullin through the action of PAD enzymes. There are 5 human PAD (PAD-2-4 and PAD-6) among which PAD-2 and PAD-4 are found in the synovium and their expression levels are correlated with the intensity of inflammation [7]. Dysregulated endogenous activity of PAD enzymes in RA acts as a promoter of the autoimmune response through citrullination [8].

The role of bacteria such as PG in the etiopathogenesis of RA has been extensively explored and debated. Those bacteria responsible for periodontitis also produce PAD enzyme capable of citrullinating proteins. In this context, PG has gained much interest as a microbial agent that can initiate RA because it produces modified proteins that appear as foreign and induce specific antibodies (ACPA) [1]*.* There is a strong correlation between the presence of ACPA and periodontitis in RA patients. The presence in the blood of PD patients of ACPA that precede the onset of RA has been put forward as an argument for a pathogenic role of ACPA that can span several years [3].

In that respect, ACPA have been recently published to contribute to the intensity of bone erosion in a rat model of arthritis as well [9]. Thus, an experimental model can recapitulate the presumed initial steps of RA pathogeny.

Anaerobic bacteria that colonize the gum cause periodontitis. Some of those are capable of citrullinating proteins. *Aggregatibacter actinomycetemcomitans* (AA) has been recently published as another bacterial candidate that triggers autoimmunity in RA [10].

Cytomegalovirus and other herpesviruses have also been found in periodontitis lesions [11]. Bacteria found in periodontitis may thus be cofactors of local HCMV infection in addition to being etiologic factors of RA. HCMV, through its action on the immune system, may also contribute to the pathogenesis of bacteria known to be involved in the development of ACPA.

## HCMV and immunity

HCMV is an endemic pathogen that infects 50–100% of the world population, depending on the socio-economic environment. HCMV is a latent β-herpesvirus that is highly adapted to its host and does not cross the species barrier. After primo-infection, it remains in a latent form and reactivates upon immunosuppression [12].

Viral agents, especially chronic or latent ones, have often been hypothesized to be causative agents or at least cofactors of the pathophysiology of rheumatoid arthritis. Several arguments such as genetic, hormonal, socio-economic, and environmental that concur with the viral ones have been put forward in support of this hypothesis.

However, countries with higher rates of HCMV latent infection do not have higher prevalence of RA whose incidence is similar throughout the world [13]. This suggests that HCMV infection is not a major etiological factor of RA. Thus, how HCMV contributes to the pathogenesis of RA has been much debated and is still questioned, although a recent meta-analysis based on serology conclusion is that “HCMV was not associated with RA” [14].

The pathogenicity of HCMV depends on the integrity of the immune system. HCMV stimulates the innate and adaptive immune system [15]. Immunity allows getting over primo-infection and keeps the virus under latency and prevents reactivation. HCMV-specific CD4 and CD8 T cell responses are the highest of all immune responses against pathogens in seropositive individuals [15]. HCMV latent infection is characterized by the presence of antibodies that witness the response of the immune system to the virus and are used as markers of the previous infection. IgG HCMV seropositivity is thus a marker of ancient infection that has become latent and reactivates infrequently at some sites [16]. Because HCMV seropositivity is frequent (50–60% of the European population), it has been difficult to understand the contribution of this virus to RA pathogenesis.

Defense against HCMV involves several components of immunity and leads to the production of pro-inflammatory cytokines. Are the viral-induced inflammation and the immune response relevant in the context of RA etiopathogenesis? High levels of cytokines in the RA joints [17] may contribute to the recruitment of immune cells specific for HCMV as well as to the reactivation of latent HCMV in situ.

Latent HCMV infection is defined by the lack of viral DNA detection. Several papers report the presence of HCMV DNA in joints that is considered as the mark of viral reactivation. Reactivation of HCMV in joints has been reported to be associated to strong local immune response such as high cytokine levels [18]. Synovial membranes of some RA patients, which are obviously sites of inflammation, have been shown to harbor HCMV DNA [19]. However, it is difficult to conclude on whether this is the result of inflammation or whether local detection of HCMV is related to the etiopathogenesis of RA.

In addition, in a similar fashion, detection of multiple viral DNA species in synovial tissue and fluid of patients with early arthritis besides that of HCMV may be explained by the recruitment, into the inflamed joints, of inflammatory cells harboring diverse viral DNA [20]. DNA in situ hybridization of RA synovial specimens showed the presence of the HCMV genome. In some cases (50% of DNA-positive samples), the presence of early HCMV antigen by immunohistochemistry was also detected suggesting viral replication [21]. However, HCMV DNA was not found more frequently in the synovial fluid of RA patients as compared with controls [22]. Thus, the presence of HCMV particles or proteins in joints of RA patients has been reported but the correlation with a pathogenic role of the virus versus reactivation due to the joint inflammatory environment or to immunosuppression cannot be made.

Since HCMV can cause various end-organ pathologies such as encephalitis, hepatitis, and colitis, it is no surprise that it does also cause arthritis in adults. Such cases have been reported notably after transplantation, which is often the cause of HCMV reactivation or of primary infection when the donor is HCMV seropositive. For example, arthritis related to HCMV infection has been reported after bone marrow transplantation [23] and kidney transplantation [24], although this is a rare event. It thus cannot be formally excluded that HCMV plays a causative role in a minority of RA patients.

As for other herpesviruses, EBV and HHV6 DNA, but not HCMV, have been found more frequently in the blood of RA compared to controls [25]. Thus, some herpesviruses may be more frequent in RA, and this may be due to immune deficiency observed in the course of the disease or to therapeutic immunosuppression.

On an epidemiologic point of view, HCMV seropositivity has not been found to be associated with RA [14] although a higher frequency of IgM anti-HCMV, as compared to IgG was found in RA patients, but not healthy controls, from Colombia [26]. This is, however, subject to caution as IgM could reflect general activation of the immune system. This points out to the requirement of a more subtle analysis of anti-HCMV immunity in RA patients. It may be worth checking for anti-HCMV low avidity IgG or HCMV DNA in the blood of recently diagnosed RA as a marker of recent HCMV infection.

As far as cellular immunity is concerned, NK cells, CD4, and CD8 T cells play a crucial role in the defense against acute and latent HCMV. CD8 T cell specificity against HCMV can represent the majority of CD8 repertoire in latently infected subjects but are in an exhausted state [15]. As a major component of anti-HCMV immunity, CD8+ T cells are present at a high frequency in the blood of seropositive individuals as well as RA patients. CD8+ T cells specific for HCMV are frequently enriched in the inflammatory lesions of RA but other diseases as well [27]. The significance of HCMV-specific T cell clonal expansions found in inflammatory sites of RA and other autoimmune diseases does not relate to any pathophysiological role but rather, as alluded to above, to a homing due to migration and trapping of responsive cells.

In patients treated with anti-TNF, anti-HCMV CD4+ [28] and CD8+ T cell responses [29] are not altered by anti-TNF treatment, suggesting that the course of the RA disease is not connected to the level of anti-HCMV response.

Expansion of CD28neg CD4 T cells is a hallmark of HCMV immune response. The accumulation of such cells in autoimmune disease such as RA may suggest a correlation [30]. In that regard, whether peptide(s) from HCMV antigens, if any, is(are) involved in the expansion of CD28 neg CD4 T cells needs to be explored. On the other hand, HCMV has been considered as the prototype of a pathogen capable of escape from the immune system [31]. Escape occurs through MHC downregulation but also modulation of the host cytokine immune response, e.g., through the production of cellular and viral IL-10 [12] inhibition of the HLA-Class II transactivator CIITA [32] and resistance to IFN-g-mediated STAT1 activation [33]. CIITA has also been involved in OC differentiation and its ablation suppressed OC differentiation [34]. Decreasing CIITA is yet another mechanism through which HCMV could inhibit OC differentiation.

Linking HCMV infection with the intensity of RA symptoms has been tempting. Indeed, Pierer et al. reported that joint destruction (as defined by synovectomies or joint surgery procedures) was more frequent in HCMV seropositive RA patients as compared to seronegative ones although there was no report of specific bone erosion [35]. It thus appears that HCMV infection may worsen the articular evolution and outcome of RA.

Regarding the etiopathogenesis of RA, it has to be noted that similar to joints, HCMV has been detected in periodontitis lesions. DNA amplification studies by Popovic et al. [11] found that HCMV was present in large, symptomatic periapical lesions. It thus appears that HCMV is related to periodontitis by itself in some PD patients. This, however, does not imply that HCMV is a direct cause of periodontitis.

Regarding the immune response to herpesviruses, EBV and HCMV were found to be associated with joint damage in RA but not with bone erosion evaluated by the Sharp-van der Heijde score (SHS) in HCMV-positive patients [36]. Thus, the relationship between HCMV seropositivity and RA is complex.

## Consequences of HCMV infection on the myeloid lineage

How HCMV may contribute to the pathogenesis of RA is thus complex. Macrophages are central to the pathogenesis of RA and are targets of HCMV as well. The virus can replicate in those cells but can also remain in a latent state and can be reactivated depending on the environment [16]. In that respect, HCMV DNA detection in joints of RA patients may be related to the presence and reactivation of HCMV in macrophages present in the joints [22].

Monocytes/macrophages play a crucial role in RA. First, as macrophages are effectors of inflammation, they participate strongly in the pathophysiology of RA and are targets of biotherapies [37]. They are responsible for bone erosions through their differentiation into OCs. Although the origin of inflammation in RA is certainly diverse, one of the elements to be considered is HCMV. Because infection of monocytes induces an inflammatory profile, HCMV is a candidate for participating in the pathophysiology of RA. HCMV uses elements of the cPLA2 inflammatory pathway to infect cells [38], and inhibition of cyclooxygenase blocks viral spread [39]. However, those elements do not provide any direct physiopathological explanation for the development of arthritis. In that respect, they may add to the local inflammatory process [35].

In RA, criteria to define and quantify bone erosion have been precisely defined by van der Heijde et al. [40]. We have recently observed that, in a large cohort of recently diagnosed RA patients, the “ESPOIR” cohort, progression of bone erosion was slower in patients seropositive for HCMV [5]. There may be several explanations for this observation: first, QKI5, an mRNA-binding protein induced by HCMV in monocytes/macrophages, may inhibit OC differentiation as proposed in our other recent publication [41]. The direct inhibition of CSF-1R by QKI5 [41] may bring at least a partial explanation to the diminished progression of erosion observed in early RA patients of the “ESPOIR” cohort [5]. Overexpression of QKI5 using lentivirus clearly led to decreased osteoclastogenesis in vitro and to reduced bone resorption in vivo in a calvaria bone model [41]. We propose the hypothesis that reduced progression of bone erosion is mediated by QKI5 in RA patients. The expression of QKI5 in RA patients needs to be investigated to confirm this hypothesis. In RA as well as other pathologies with bone erosion, methods to increase expression of QKI5 in patients as a way to reduce OC activity are worth exploring as a novel therapeutic concept. Using QKI-deficient mice, Du et al. have recently reported a role of QKI in RANKL-induced osteoclastogenesis [42].

There have been recent reports about the modulation of macrophage properties by QKI5 as reviewed by Darbelli et al. [43]. Differentiation of monocytes into macrophages is associated with an increase of QKI proteins, and in a QKI-haploinsufficient patient, a reduction of QKI expression limits monocyte to macrophage differentiation. Overexpression of QKI5 orientates towards an M2 phenotype whereas quaking deficiency amplifies inflammation as observed in experimental endotoxemia. QKI5 also participates in the regulation of multiple cell types. Other aspects such as macrophage polarization in the presence of QKI5 should bring more information about its role in inflammation and its resolution.

Thus, multiple mechanisms can explain the inhibition of OC differentiation by HCMV. When infecting monocytes, HCMV induces their differentiation into macrophages and reprograms them towards an M1-like phenotype. Because those M1-like macrophages produce IFN-a and IL-10, this pathway of differentiation and subsequent polarization may by itself inhibit osteoclastogenesis [44].

Polarized M1 macrophages can be infected by HCMV and trigger inflammation, but IL-10 and IL-4 produced by infected M2 macrophages may be inhibitory factors of osteoclastogenesis as well. Viral IL-10 produced by infected macrophages that induces “M2c” macrophages also inhibits OC differentiation [45].

Activation of TLRs by binding of HCMV to cells [46] is another cause of inhibition of OCs due to signaling through NF-kB and inhibition of CSF-1R and RANK [47]. All those factors could concur to the inhibition of bone erosion in a somewhat contra-intuitive way. However, none of them is incompatible with the role of QKI5, either in M1 or in M2 macrophages.

## Cooperation bacteria/HCMV

Although HCMV by itself is not capable of citrullinating proteins, it may, through interactions with bacteria, be relevant to the pathogenesis of RA. This has however never been formally demonstrated although RA and PD have been hypothesized to share pathogenic factors such as herpesviruses and bacteria [3].

On the one hand, bacterial pathogens are proposed as causative agents of RA. Since, on the other hand, no direct contribution of viruses to RA has been found so far, a combination of viral/bacterial factors could be accountable for the origins of the disease.

The inflammatory environment due to local bacteria may be factors of local HCMV reactivation. Besides, the production of pro-inflammatory cytokines may be increased by the bacteria-virus coinfection, be it simultaneous or sequential, and induce the local destruction of adjacent tissue. Could that potentiation have an impact on RA etiopathogenesis? Mutual potentiation of virus and bacteria in periodontitis has been reviewed [48]. In addition, significant associations of HCMV and PG or AA with progressive periodontitis have been extensively reviewed by Chen et al. [48].

Interaction between bacteria and HCMV has been proposed to explain the breakdown of periodontal tissues. HCMV was positively associated with coinfections of PG and *P. nigrescens* (OR 3.23) [49]. This suggests that HCMV may promote gingival colonization of AA and PG. At the cytokine level, mechanisms of bacteria/HCMV cooperation may involve potentiation of IL-1β production as proposed by Wara-Aswapati et al. [50]. However, although the relationship between the presence of periodontal herpesviruses and PG was observed, this is not proof that they are cofactors of PD.

The way viruses may help bacterial colonization are various. For example, respiratory viruses promote bacterial adhesion in a cell-specific way [51] and infection with influenza A virus results in the induction of invasive *S. pyogenes* infection in mice [52]. However, similar cooperation has still to be reported for HCMV and periodontitis bacteria. Thus, although HCMV does not appear to be a direct causative agent of RA, it may favor the lesions due to PG and AA in periodontitis that are sources of PAD and associated with the etiopathogenesis of RA. At the molecular level, several mechanisms such as escape from the immune response by HCMV may favor local immune suppression that can help bacterial growth in PD lesions.

Future investigations will search for formal molecular proof of potentiation of HCMV by bacteria or vice versa. This could lead to ways of blocking this cooperation in order to dampen inflammation (Table 1).

**Table 1:** Summary of key points discussed in the text

• HCMV infection does not appear to be a causative agent of RA.	
• HCMV may increase local inflammation caused by *P. gingivalis* in periodontitis.	
• This increase may occur either through immune response or through intrinsic action.	
• RA patients seropositive for HCMV display lower bone erosion.	
• HCMV inhibits OC differentiation through the induction of QKI5.	
• HCMV may also participate in lowering bone erosion through the induction of IFN-α and IL-10 and by producing viral IL-10. IFN-γ produced during the immune response may be involved in this inhibition process.	
• Our hypothesis is that HCMV is an aggravating agent of RA on the one hand while seropositivity reduces bone erosion possibly through QKI5 expression on the other hand, although a combination of other mechanisms certainly applies.	

## Conclusions

**Fig. 1:**
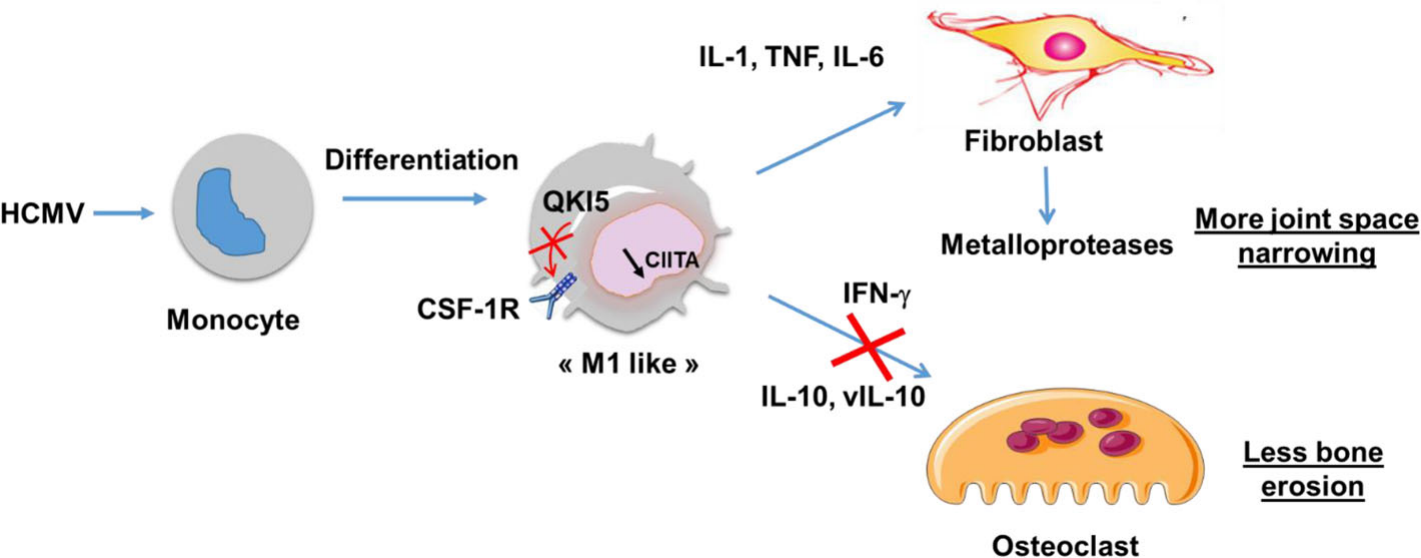
Overview of the mechanistic role of HCMV in the articular damage in RA. Infection of monocytes by HCMV induces QKI5 that inhibits the expression of CSF-1R and inhibits CIITA. Consequently, OC differentiation is inhibited. Infected monocytes differentiate into “M1-like” macrophages that produce pro-inflammatory cytokines and help fibroblasts produce metalloproteases. Production of viral IL-10 may contribute to perpetuate to decreased OC differentiation. Contribution to the inhibition of OC differentiation may be due to IFN-γ produced by “M1-like” macrophages

In conclusion, we propose that HCMV has a dual role in the pathogenesis of RA: aggravating inflammation in PD as well as in joints while protecting from bone erosion through various mechanisms, as summarized in Fig. 1. Joint destruction results in physical disability, and in this process, cartilage damage is more invalidating than bone erosion and the two may evolve separately [53]. Thus, in this regard, HCMV infection may be considered as a foe in the course of RA. However, studying HCMV has pointed to the possible use of QKI5 as a cellular target for the treatment of bone erosion in various pathological contexts.

## Data Availability

Not applicable

## Abbreviations

HCMV: Human cytomegalovirus
RA: Rheumatoid arthritis
ACPA: Anti-citrullinated protein antibodies
PG: *Porphyromonas gingivalis*
AA: *Aggregatibacter actinomycetemcomitans*
PD: Periodontal disease
